# Optimal Design of Nanoparticle Enhanced Phan-Thien–Tanner Flow of a Viscoelastic Fluid in a Microchannel

**DOI:** 10.3390/e20120895

**Published:** 2018-11-22

**Authors:** Mohammad Yaghoub Abdollahzadeh Jamalabadi

**Affiliations:** 1 Department for Management of Science and Technology Development, Ton Duc Thang University, Ho Chi Minh City 700000, Vietnam; abdollahzadeh@tdtu.vn.edu; Tel.: +82-010-7435-1362; 2 Faculty of Civil Engineering, Ton Duc Thang University, Ho Chi Minh City 700000, Vietnam

**Keywords:** nanofluids, entropy generation minimization, microchannel, heat transfer

## Abstract

The excellent thermal characteristics of nanoparticles have increased their application in the field of heat transfer. In this paper, a thermophysical and geometrical parameter study is performed to minimize the total entropy generation of the viscoelastic flow of nanofluid. Entropy generation with respect to volume fraction (<0.04), the Reynolds number (20,000–100,000), and the diameter of the microchannel (20–20,000 μm) with the circular cross-section under constant flux are calculated. As is shown, most of the entropy generation owes to heat transfer and by increasing the diameter of the channel, the Bejan number increases. The contribution of heat entropy generation in the microchannel is very poor and the major influence of entropy generation is attributable to friction. The maximum quantity of in-channel entropy generation happens in nanofluids with TiO_2_, CuO, Cu, and Ag nanoparticles, in turn, despite the fact in the microchannel this behavior is inverted, the minimum entropy generation occurs in nanofluids with CuO, Cu, Ag, and TiO_2_ nanoparticles, in turn. In the channel and microchannel for all nanofluids except water-TiO_2_, increasing the volume fraction of nanoparticles decreases entropy generation. In the channel and microchannel the total entropy generation increases by augmentation the Reynolds number.

## 1. Introduction

Heat transfer exchange in fluid flows [1] has numerous modern and common applications, including electronic cooling [2], mass transport [3], life [4], and cooling frameworks with phase change materials [5]. Conventional liquids, for example, water, oil, and glycols have poor heat transfer exchange because of their low heat transfer conductivities [6]. Innovative work has been completed to enhance the heat transfer improvement of liquid. Strong metallic materials and non-metallic materials have considerably higher heat transfer conductivity than the base liquid. A nanofluid is defined as a suspension of nanosized metal molecules smaller than 100 nm in the base liquid [7]. Nanofluids are drawing extraordinary intention with their tremendous potential to improve execution properties, particularly with the regard to warm exchange [7]. Nanofluids as a combination of metal, polymer, or metal oxide in a Newtonian liquid (mostly water or alcohol) present specific features in various fields of engineering [1,2,3,4,5,6,7] and biology [8,9,10,11,12,13,14]. In the field of heat transfer, the higher value of thermal conductivity in nanofluids provided by solid nanoparticles increases convective heat transfer in comparison with ordinary liquids. The heat dissipation required for some supercomputers is approximately 1 megawatt per square meter which should be cooled by liquid nitrogen [15]. For refrigeration of these circuits, air cooling systems are not enough and nanofluids can be suitably replaced for cooling them [16]. 

The efficiency of a process can be evaluated by entropy analysis. Irreversibility of such systems can be calculated with the aid of the second law of thermodynamics (T_0_S_gen_) [17]. If the amount of system entropy generation throughout a heat dissipation process with simultaneously higher heat transfer and higher pressure drop (general effect of nanoparticles adding) is unknown, a detailed second law analysis is required. The higher heat transfer guarantees the reduction of irreversibility while pressure drop increase leads to an increase of the of system irreversibility. This compromise leads to an optimal entropy generation point. As stated by the main beliefs of entropy minimizing theory [18], the optimum state of a thermal system is at the minimum entropy generation point.

In other verses, the best strategy for a microchannel heat exchanger is reducing the pressure drop and increasing the thermal efficiency simultaneously [19]. Meanwhile a few works on Phan-Thien–Tanner (PPT) nanofluid turbulent flow entropy generation in microchannels and channels has been completed [20], the generation of entropy in the microchannel and channel is better thought-out even though the cited articles may be related to nanofluids or non-nanofluids with regards to laminar or turbulent flow [21]. 

Mahmoud and Fraser [6] examined entropy generation and heat transfer of fluid flow confined to the porous medium channel. The channel walls are supposed to be kept at different temperatures and the fluid flow is laminar. At a constant Darcy number and porosity it shows that the Bejan number increases to around channel half-width; afterwards it decreases. Koh and Chenge [21,22] offered a numerical work in laminar entropy generation of a wavy channel fluid flow. They stated that minimal entropy generation occurs at minimum wavy wall amplitude. Tshehla and Makinde [23] inspected the fluid entropy generation between concentric circular cylinders. It was shown that entropy generation is proportional to the Brinkman number (Brinkman number in polymer processing is a dimensionless number related to heat conduction from a wall to a flowing viscous fluid). Bianco et al. [24] explored entropy generation and turbulent flow heat transfer of nanofluids of water-Al_2_O_3_ in a square cross-section channel numerically. They testified that at a constant volume fraction, heat entropy generation decreases and friction entropy generation increases by growing the Reynolds number. They demonstrated that there is an optimum Reynolds number. Glycol-Al_2_O_3_ and water-TiO_2_ nanofluids at the laminar and turbulent flow in a channel are the subjects of Mahian et al. research [25]. Agreeing to previous outcomes by increasing the volume fraction, the entropy generation decreases. 

Mahian et al. [26] studied the effects of different thermophysical models properties for nanofluids of ethylene glycol-Al_2_O_3_ on the generation of entropy between coaxial cylinders. Founded on their consequences, the critical radius is predicted (same for all different thermophysical models) and the predicted entropy generation decreases by increasing the nanoparticles volume fraction. Leong et al. [27] inspected water-TiO_2_ and water-Al_2_O_3_ nanofluids and showed that the TiO_2_ entropy generation is less than Al_2_O_3_. Mahian et al. [28] studied the effect of magnetic fields on the entropy generation of water-TiO_2_ nanofluids in a space between two coaxial channels. They suggested that in the presence of the magnetic field, nanofluids are only used for small amounts of the Brinkman number in order to justify increasing heat transfer and entropy generation. In contrast, Bianco et al. [29] found the minimum entropy generation at low volume fractions of nanoparticles. They investigated water-Al_2_O_3_ nanofluids in a circular cross-section pipe numerically. Microchannel laminar and turbulent flow of water-Al_2_O_3_ is examined by Singh et al. [30]. They illustrated that in turbulent flow, Al_2_O_3_ reduces the generation of entropy. In addition, a water-CuO nanofluid in a trapezoidal microchannel was the topic of study for Li and Kleinstreuer [31]. They surveyed the laminar entropy generation and found an optimal volume fraction of CuO nanoparticles. Mah et al. [32] analytically inspected the water-Al_2_O_3_ viscosity effects. They found entropy generation increases by volume fraction. Elazhary and Soliman [33] showed heat transfer is dominant in entropy generation of fully developed laminar forced convection in a parallel plate microchannel. Additionally, they found that the use of fins improves heat transfer and reduces the entropy generation as shown by Zhai et al. [34]. 

Fabrication technology and thermal hydraulic performance of microchannel flow passages are comprehensively reviewed by Kandlikar [35]. From the time of Maxwell’s work [36] to now various models for nanofluids have been reported [37]. A comprehensive review of heat transfer and flow performance of nanofluids in microchannel heat sinks shows that the simple classification of heat sink groups are passive (economic and accessible but power limited), semi-active, active (combined heat sink and fan), liquid cooling (complicated and expensive but efficient), and phase change cooling (equal heat dispersion) [37]. Pourmehran et al. [38], Hatami and Ganji [39], Sakanova et al. [40], and Ting et al. [41] worked on a fin-shaped microchannel heat sink cooled by different nanofluids concentration. As of late, there have been few examinations which concentrated on the entropy rate of the nanofluid stream [42,43]. Leong et al. [42] and Bianco et al. [24,29] directed an entropy rate production examination on nanofluid (alumina/water) in round tubes and square section tubes respectively.

There have already been several viscoelastic constitutive models of the polymer, for example, Chilcott–Rallison, Phan-Thien–Tanner, and Oldroyd-B. Although the PTT is a pure rheological model and model complexity is high, its parameters are measurable via experiments [43]. The PTT class of models offers a better alternative in the modeling of the differential type models which used by other authors. While the Giesekus model has one parameter for nonlinearity, the PTT model has two. Moderately the PTT model has the ability to describe elongational and shear properties independently. 

Considering the preceding studies, it is obvious that no studies have been completed about the in PPT fluid flows in channels and microchannels. Additionally, based on the author’s knowledge up to now, no experimental studies of nanofluids reported such a behavior. The main components (or the key parameters) involved in these entropy generation types of studies are fluid characteristics and the thermal properties of the fluid which in this study is PPT fluid where has not discussed before. This work uses numerical modeling of nanofluids with PTT governing equations to calculate the optimal design of a system based on the current knowledge. This study, on the effect of nanoparticles on entropy generation of PPT nanofluids by solution of continuity and Navier–Stokes equations, includes the effect of linearized thermophysical expressions. Then a parameter study is done to evaluate the entropy generation of flow by considering the Reynolds number, type of nanofluids, the volume fraction, and the diameter for circular cross-section microchannels under a constant heat flux.

## 2. Materials and Methods 

**Figure 1:**
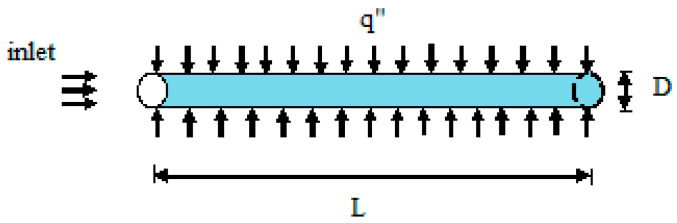
Schematic of microchannel configuration.

To accomplish the goals of this examination, the entropy rate investigation for mini-channel and microchannel have assessed. The count part of this investigation is isolated into two areas and each segment has subsection. Entropy rate conditions for the microchannel and mini-channel were as described previously by Kandlikar [35]. The impact of various nanoparticles for entropy rate in microchannel and mini-channel with various base liquid is related with the expansion in rate volume part running from 0% to 4%. In Figure 1 a lateral view of the channel with length L (D is diameter were according to Kandlikar [35] channels classified based on the diameter as a normal channel ($D_{h}\geq 3\;\mathrm{mm}$), mini-channel ($200\;\mu m\leq D_{h}\leq 3\;\mathrm{mm}$), and microchannel ($10\;\mu m\leq D_{h}\leq 200\;\mu m$) is shown. This study has been done for nanofluids and polyisobutylene (PIB) solutions in Decalin which is used as a base fluid for nanoparticles of Ag, Cu, CuO, and TiO_2_, Reynolds numbers of 20,000, 40,000, and 100,000, volume fractions of 0, 0.01, 0.02, 0.03, and 0.04, and microchannel diameters of 20 to 20,000 micrometers. The turbulent flow in the channel is fully developed. Thermophysical properties of fluid and nanoparticles are given in Table 1. To extract the nanofluids properties [31] linear effective and Maxwell’s relation were used [36]. The thermal conductivity, nanofluid viscosity, nanofluid density, and nanofluid specific heat, are obtained using

(1) $$\rho_{nf}=(1-\varphi )\rho_{f}+\varphi \rho_{s}\;$$

(2) $${\left(\rho c_{p}\right)}_{\mathrm{nf}}=(1-\varphi ){\left(\rho c_{p}\right)}_{f}+\varphi {\left(\rho c_{p}\right)}_{s}\;$$

(3) $$\mu_{\mathrm{nf}}=\mu_{f}{\left(1-\varphi \right)}^{-2.5}\;$$

(4) $$\;\frac{k_{\mathrm{nf}}}{k_{f}}=\frac{\left(k_{s}{+2k}_{f}-2\varphi \left(k_{f}{-k}_{s}\right)\right)}{\left(k_{s}{+2k}_{f}+\varphi \left(k_{f}{-k}_{s}\right)\right)}\;$$

The Equation (1) is computed by Maxwell effective medium theory. The forecasted effective thermal conductivity of nanofluid can be strong-minded when the particle’s thermal conductivity is much higher than the thermal conductivity of the base fluid. The differential energy absorbed for dx length element by $q^{\prime }$ heat transfer rate per unit length in the tube leads to a ‘*dh’* change in nanofluids enthalpy ($q^{\prime }\mathrm{dx}=\dot{m}\mathrm{dh}$). Entropy generation is composed of two parts: pressure drop, ${S^{\prime }}_{\mathrm{gen},\Delta P}^{}$ and heat transfer, ${S^{\prime }}_{\mathrm{gen},\Delta T}^{}$, which can be expressed as

(5) $${S^{\prime }}_{\mathrm{gen}}^{}=\dot{m}\frac{ds}{dx}-\frac{q^{\prime }}{T+\Delta T}={S^{\prime }}_{\mathrm{gen},\Delta T}^{}+{S^{\prime }}_{\mathrm{gen},\Delta P}^{}\;$$

where $S^{\prime }$ is entropy generation per unit length and ΔT is temperature difference between the tube wall and the bulk flow of nanofluids. Based on Maxwell’s relation entropy change is obtained by

(6) dh=Tds+νdp 

By substituting ($ds=\frac{\mathrm{dh}-\nu \mathrm{dp}}{T}=\frac{q^{\prime }\mathrm{dx}}{\dot{m}T}-\frac{\nu \mathrm{dp}}{T}$) in Equation (5) at ΔT+T:

(7) $${S^{\prime }}_{gen}^{}=\frac{q^{\prime }}{T}-\frac{q^{\prime }}{T+\Delta T}-\frac{\nu }{T}\frac{dp}{dx}=\frac{q^{\prime }\Delta T}{T^{2}(1+\frac{\Delta T}{T})}+\frac{\dot{m}}{\rho \;T}(\frac{-dp}{dx}))≅\frac{q^{\prime }\Delta T}{T^{2}}+\frac{\dot{m}}{\rho \;T}(-\frac{dp}{dx})\;$$

The first and second sentences indicate the entropy generation due to heat transfer and pressure drop, respectively. Equation (7) can be rewritten based on dimension parameters such as the Stanton number. Correlations for the Stanton number, friction factor, Weissenberg number, and Nusselt number proposed are as follows

(8) St=hnfρnfunfcp,nf=q′/ΓΔTcpG=NuRePr, 

(9)  Re=4m˙πμD 

(10) $$f=\frac{2D_{h}\Delta p}{\rho_{nf}u_{in,nf}^{2}L_{ch}}\;$$

(11) $$h_{ave}=\frac{q_{w}A_{film}}{A_{con}(T_{w}-T_{f})}\;$$

(12) $$Nu=\frac{h_{ave}D_{h}}{\lambda_{f}}\;$$

(13) $$Wi=\lambda \frac{U_{in}}{R}\;$$

The friction factor can be derived from the turbulent correlation:

(14) f=2ρDhG2(−dpdx)=(0.79 ln(Re)−1.64)−2 

Additionally, the Nusselt number relationship presented is the heat transfer coefficient of the turbulent flow. Here Nusselt number comes from the turbulent correlation as

(15) Nu=(f8)(Re−1000)Pr1+12.7(f8)0.5(Pr23−1), 

For an internal flow with diameter *D*, under heat flux *q”*, the total entropy generation per unit length (${\left({\dot{S}}_{gen}^{\prime }\right)}_{\mathrm{heat}\;\mathrm{transfer}}+{\left({\dot{S}}_{gen}^{\prime }\right)}_{\mathrm{fluid}\;\mathrm{friction}}$) is

(16) S˙gen′=q″2πD2kT2Nu((Re)D,Pr)+8m˙3π2ρ2Tf((Re)D)D5

where $\dot{m}=\frac{\rho_{n,f}U\pi D_{h}{}^{2}}{4}$, $G=\frac{\dot{m}}{A}{,}_{}D_{h}=\frac{4A}{\Gamma }$. This relationship only applies to specific cases of turbulence. 

The Bejan number is defined as the ratio of entropy generation due to heat (first term in the right side of Equation (7)) to the total entropy generation as

(17) $$\mathrm{Be}=\frac{S_{\mathrm{gen},\Delta T}}{S_{\mathrm{gen}}}\;$$

The Bejan number is the dimensionless pressure drop along a channel while it plays the same role in forced convection that the Rayleigh number plays in natural convection. The governing equations of the PPT viscoelastic flows are the continuity equation (conservation of mass)

(18) ∇⋅u=0, 

and the momentum equation (conservation of linear momentum)

(19) $$\rho \left(\frac{\partial u}{\partial t}+u\cdot \nabla u\right)=-\nabla p+\mu_{S}\nabla^{2}u+\nabla \cdot \sigma ,$$

where the viscoelastic extra-stress tensor (σ) is calculated by

(20) $$\frac{\partial \sigma }{\partial t}+u\cdot \nabla \sigma -\left(\sigma \cdot \nabla u+\nabla u^{T}\cdot \sigma \right)+\frac{f(\sigma )}{\lambda }\sigma =\frac{2\mu_{P}}{\lambda }\dot{\varepsilon },$$

where $\dot{\varepsilon }=\left(\nabla u+\nabla u^{T}\right)/2$ is the strain rate tensor,

(21) $$\mu_{p}=\frac{\eta_{p}}{\eta }=1-\mu_{s}\;$$

and *f*(σ) is a relaxation function which here in linear PTT

(22) $$f=1+\varepsilon \frac{\sigma_{x}^{*}+\sigma_{y}^{*}}{2}\;$$

The expressions of the relaxation function *f*(σ) in the generic constitutive form for different viscoelastic models are shown in Table 2.

## 3. Results

**Figure 2:**
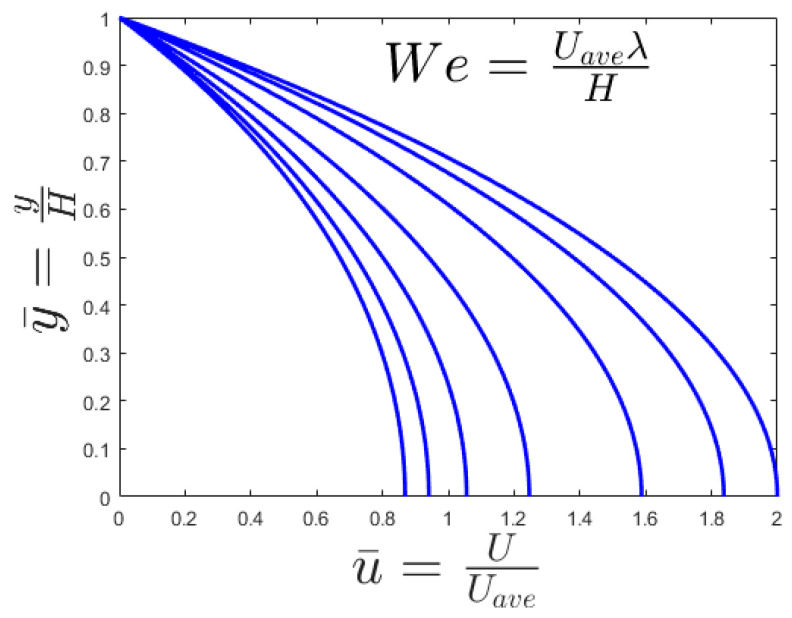
Effect of We on velocity profile.

**Figure 3:**
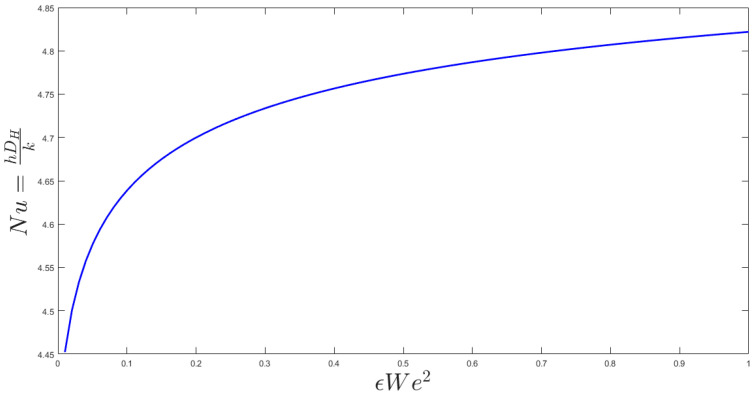
Effect of εWe^2^ on Nusselt number.

**Figure 4:**
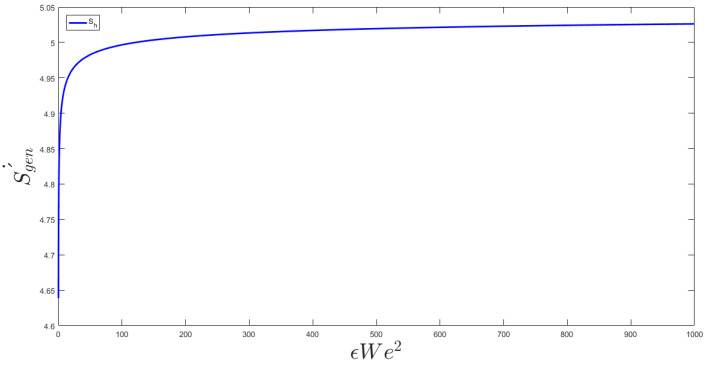
Effect of εWe^2^ on entropy generation.

The two key parameters of the problem are the Weissenberg number and Reynolds number. The Weissenberg number relates the viscoelastic effect due to inertial effects and the Reynolds number differentiates between laminar and turbulent flows. In Figure 2, the different We number is applied to the channel flow to find the velocity profile of the channel. The normal parabola shape of the velocity profile changes while the We number increase; causing an increase of the nonlinearity of the fluid viscosity. The resulted Nusselt number is illustrated in Figure 3. As shown, εWe^2^ increases as the Nusselt number increases to a maximum Nusselt number. There is a limiting value of the Nusselt number in Figure 3 which shows that the maximum heat transfer in the system cannot increase too much by an increase of its nonlinearity. Figure 3 describes the quantitative values of entropy generation by frictional and thermal terms for Nanofluids in microchannel flow. The same phenomena could be seen in the entropy generation figure. Figure 4 presents the effect of εWe^2^ on entropy generation. As shown, εWe^2^ increases entropy generation to a maximum Nusselt number. The limiting case here also reached as the heat transfer reaches its maximum. For the conventional channel, the curves show that the entropy generation rate is below 5.5 for all the range.

**Figure 5:**
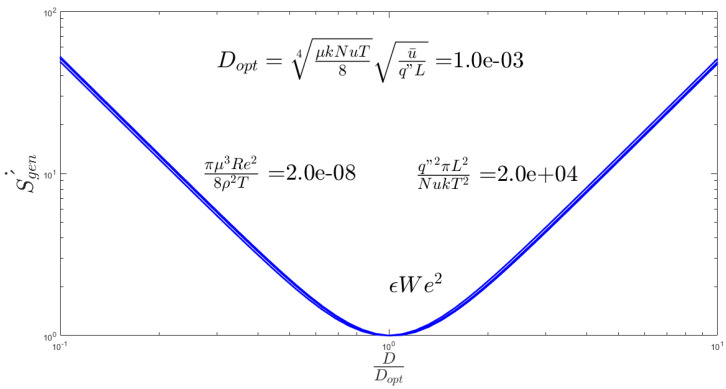
The total entropy generation variations in terms of the channels diameter for water and different nanofluids in Re = 20,000.

Figure 5 plots the total entropy generation variations in terms of the channel’s diameter for water and different nanofluids in Re = 20,000. Optimal diameter based on entropy generation is

(23) $$D_{optimal}=\sqrt[{4}]{\frac{\mu kTNu}{8}}\sqrt{\frac{\bar{u}}{q^{\prime \prime }L}}$$

**Figure 6:**
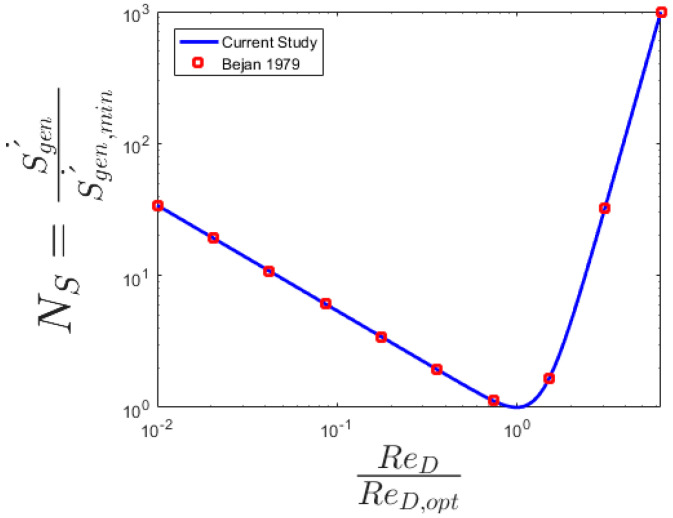
The entropy generation number variations in terms of the Re (optimal Re based on entropy generation).

The point is found by the scale analysis of the two components of entropy generation. It is good to notice that for a simpler problem of laminar flow of Newtonian fluid the heat transfer and friction coefficients are constant as Nu=4811 and f=64Re. Since the total entropy generation is

(24) $${\dot{S}}_{gen}^{\prime }=\frac{11}{48}\frac{q^{\prime \prime 2}\pi D^{2}}{T^{2}}\frac{1}{k}+\frac{128{\dot{m}}^{2}}{\pi \rho^{2}TD^{4}}\mu \;$$

and the optimal point of total entropy generation could be found as a function of diameter. Figure 6 presents the entropy generation number variations in terms of the Re. Optimal Re based on entropy generation is found analytically from the relation

(25) Reoptimum=ρu¯6kTNu8q″2L2μ34

where $\bar{u}$ is the average velocity. Figure 6 depicts the entropy rate proportion for turbulent stream and demonstrates the conduct only inverse to laminar stream. It may be seen that entropy proportion for all models is above the 1 level. It clarifies the handiness of nanofluids for turbulent streams in microchannels. This is certainly not an exceptionally promising perception, since it is hard to get a tempestuous stream in microchannels. At higher widths the difference in inclines in entropy age proportion is low. Additionally, the impact of focus on entropy age rate proportion is more at higher distances across.

**Figure 7:**
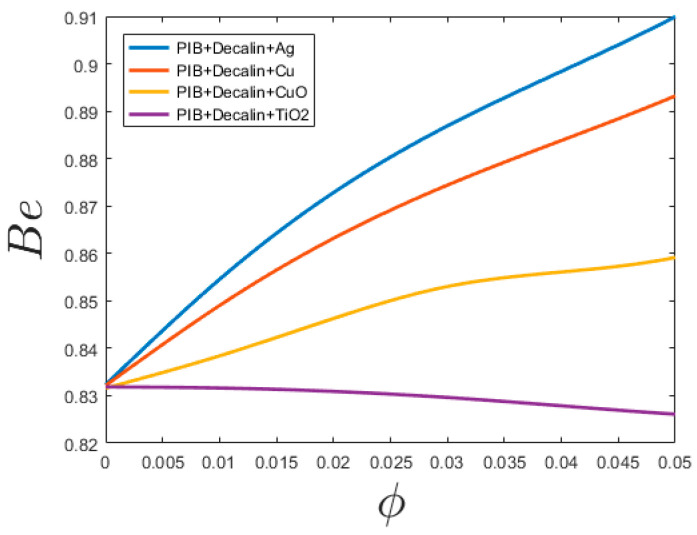
Bejan number for Phan-Thien–Tanner (PTT) nanofluids with different nanoparticles volume fractions at Re = 20,000.

Figure 7 is plotted to show the relative importance of flow frictional and thermal entropy generation rates. Thermal irreversibility is distinct over and done with a dimensionless Bejan number (Be) is shown as

(26) $$Be=\frac{{\dot{S}}_{gen\Delta T}^{\prime }}{{\dot{S}}_{gen\Delta T}^{\prime }+{\dot{S}}_{gen\Delta p}^{\prime }}\;$$

Hence, Be signifies the fraction of thermal entropy generation. Figure 7 plots the Bejan number for PTT nanofluids with different nanoparticles volume fractions at Re = 20,000. The results show the increase of Bejan number, which is the symbol of thermal components in entropy generation. The exception is PIB-Declain-TiO_2_ as presented in Figure 7 where the fluid component increases its contribution to entropy generation slightly. It is caused by the increase in viscosity of the system that is greater than the increase in thermal diffusivity. It can also be seen that the contribution of thermal entropy generation decrease with volume fraction. It implies that for the heat part, nanofluids lessen the entropy rate, yet the commitment of frictional stream entropy age part is considerably more and heat transfer part does not have any effect on generally exergetic adequacy. But at lower diameters or in microchannel regimes, friction losses convert more imperative and contribute more to the whole entropy generation rate and so decrease the Be. Despite the fact that the pattern of Be is same for both laminar and tempestuous stream with volume portion, the distinction can be found in the greatness of Be. At the microchannel level Be is higher for a laminar stream when compared with a turbulent stream. This suggests that the stream frictional entropy rate commitment is decreasing relative to the turbulence stream when contrasted with that of the laminar stream. This further demonstrates the idea that the impact of consistency is milder in the laminar stream.

## 4. Discussion

To begin with, it is of key significance to comprehend the effect of nanoparticles on the turbulent regime. As commented previously [35], the energy cascade can suppress turbulent fluid flow. That is, the motor vitality started by the disturbance enters bigger whirlpools then is exchanged to smaller swirls and at that point, into further smaller swirls, until the point that it is converted to warm by gooey powers. These actualities are key to comprehend if the nanoparticles can struggle with this vitality trade, along these lines stifling choppiness. Until the length scales of the turbulent eddy are much larger than the nanoparticle size, the nanoparticles are transported very effectively by the turbulent flow. Length scales of turbulent eddies is found from

(27) ls≈DRe−3/4 

Increasing the Reynolds number causes the heat transfer entropy rate to diminish and, the frictional and aggregate entropy rate to expand. Thermal losses diminish by expanding the nanovolume portion. The nanoparticles in the base fluid decreased the heat transfer entropy rate in spite of the fact that nanoparticles enhanced the total entropy generation. The lowest minimal heat transfer entropy rate was accomplished for ϕ = 0.04. It is noticed that the microchannel diameter was profoundly powerful on heat transfer entropy rate. Heat transfer entropy rate diminished when the microchannel diameter decreased.

In the frictional entropy rate for various volume portions, no momentous contrast was watched. Lessening the microchannel diameter caused the frictional entropy rate to increment. The frictional irreversibilities expanded by diminishing channel diameter. The fluid flow equation ($f=1+\frac{1-\xi }{2-\xi }\varepsilon \sigma_{x}^{*}$) in the simplest case has a solution of

(28) $$u_{y}|{}_{\xi =0}=\gamma =\frac{1}{\lambda \left(2-\xi \right)}\left(\tau^{*}+\frac{1-\xi }{2-\xi }\varepsilon \tau^{*}{}^{3}\right)\;$$

which leads to

(29) $$u=\frac{p_{x}}{\eta }y^{2}+\frac{\varepsilon \lambda^{2}p_{x}^{3}}{\eta^{3}}y^{4}+u_{\max}=\frac{p_{x}}{\eta }\left(y^{2}-H^{2}\right)+\frac{\varepsilon p_{x}^{3}\lambda^{2}}{\eta^{3}}\left(y^{4}-H^{4}\right)\;$$

Since the Results of Figure 2 is justifiable. As calculated for Figure 5 the optimal diameter for the ordinary conditions is 1 mm. The general trend is the same as Figure 8 of [24] for total entropy generation. Since for channels with diameters more than 1 mm (millichannels) by increase the D, the generated entropy increase while for channels with diameters less than 1 mm (microchannel) by increase the D, the generated entropy decreases. The strongest change in the total entropy generation by increasing the channel diameter can take place for water-TiO_2_ because it has the highest C_p_. Bejan number is the measure of heat transfer part. Since the results of Figure 7 show that the main part of generated entropy is attributable to heat transfer. The increases of Bejan number by increasing the channel diameter is due to the fact that the heat part is not a function of D while the viscous part decreases dramatically by an increase of D (see Equation (16)). Between the PPT-nanofluids that are studied for all diameters, the maximum Bejan number is for PIB-Decalin-Ag and the minimum Bejan number is for PIB-Decalin-TiO_2_ [35,42]). As stated, since the heat part is not a function of tube diameter and the viscous part increase dramatically by a decrease of tube diameter, the entropy generation in the microchannel is mostly due to the viscous dissipation. Entropy generation in the channel is proportional to the C_p_. As nanoparticles in the range of C_p_ can be ordered as Ag, Cu, CuO, to TiO_2_, the maximum irreversibility occurs respectively. Despite the fact, this behavior is in the microchannel represents the lowest entropy generation in that order. As well the entropy generation reduces by increasing the volume fraction and the Reynolds number. Smaller diameter showed less entropy generation in case of all nanofluids.

## 5. Conclusions

The present paper researches the entropy age examination of nanofluids. For this, model of Phan-Thien–Tanner has been considered, which speak to the hypothetical and exploratory qualities. This study, explores the effect of nanoparticle on entropy generation of the PPT nanofluids, by solution of the continuity and Navier–Stokes equations including the effect of linearized thermophysical expressions. Then a parameter study is done to evaluate the entropy generation of flow by considering the Reynolds number, type of nanofluids, the volume fraction, and the diameter for circular cross-section microchannels under a constant heat flux. It turns out from the examination that the preferred standpoint or impediment of various nanofluids can be anticipated for microchannel and customary channels with turbulent stream by taking an estimate dependent on the size of particles. It is shown that there is an ideal diameter at which the entropy age rate is the minimum. For every other case, channel heat entropy rate dependably diminishes with volume portion, while the gooey part dependably increases with volume division. It has likewise been demonstrated that at less distance across thick entropy rate part is more essential and at higher measurements heat part contributes more to entropy rate. At last, it is demonstrated that after a specific distance across the entropy rate proportion winds up consistent or increments gradually. The main conclusion remarks are as follows.

- Total entropy generation rate increases with the increase of temperature.
- Metal nanoparticles have substantially higher conductivity than liquids, thus suspending particles can build the conductivity of the base liquids and utilization of nanofluids increment the performance of miniaturized scale channel sinks.
- The entropy generation rate of fluid friction decreased productively by the improvement of volume fraction of the nanoparticles.
- The optimal diameter for the ordinary conditions is 1 mm.
- The contribution of thermal entropy generation decrease with volume fraction.
- The sturdiest change in the total entropy generation by increasing the channel diameter can take place for water-TiO_2_ because it has the highest C_p_.
- In millichannels the main part of generated entropy is attributable to heat transfer.
- In millichannels the maximum irreversibility occurs in PIB-Decalin-Ag, PIB-Decalin-Cu, PIB-Decalin-CuO, and PIB-Decalin-TiO_2_.
- In microchannels the main part of generated entropy is attributable to fluid flow.
- In microchannels the minimum irreversibility occurs in PIB-Decalin-Ag, PIB-Decalin-Cu, PIB-Decalin-CuO, and PIB-Decalin-TiO_2_.

## Figures and Tables

**Table 1:** Thermophysical properties of the base fluid (at 300 K) and nanoparticles [35].

Thermophysical Properties	1.3% PIB in Declain	Ag	Cu	Cuo	TiO_2_
$\;c_{p}(J/\mathrm{kg}\cdot K)$	1657	235	385	535.6	686.2
*ρ* (kg/m^3^)	896.1	10500	8933	6320	4250
*k* (W/m·K)	0.132	429	401	76.5	8.95
*β* × 10^−5^ (1/k)	21	1.89	1.67	1.8	0.9
*μ* (pa.s)	0.003097 {ε = 0.04, ξ = 0.01};	-	-	-	-

**Table 2:** Expressions of the relaxation function *f*(***σ***) in the generic constitutive Equation (20), for different viscoelastic models.

Viscoelastic model	Relaxation functionf(σ)
Oldroyd-B	1
Giesekus	$1+\left(\alpha \lambda /\mu_{1}\right)\sigma$
Linear PTT	$1+\left(\varepsilon \lambda /\mu_{1}\right)\mathrm{tr}\left(\sigma \right)$
Exponential PTT	$\exp\left[\left(\varepsilon \lambda /\mu_{1}\right)\mathrm{tr}\left(\sigma \right)\right]$
FENE-CR	${\left[1+\left(\lambda /\mu_{1}L^{2}\right)\mathrm{tr}\left(\sigma \right)\right]}^{-1}$
